# Supplementary material for: Acidity, sugar, and alcohol contents during the fermentation of Osmanthus-flavored sweet rice wine and microbial community dynamics
Source: PeerJ. 2025 Jan 30;13:e18826. doi: 10.7717/peerj.18826 (PMC11787802; doi:10.7717/peerj.18826)
Supplement: Supplemental Information 1 [file peerj-13-18826-s001.docx]

| **Table S1 Function Channel Statistics Table** | | | | | | |
| --- | --- | --- | --- | --- | --- | --- |
| pathway number | first-level | second-level | AG0 | AG24 | AG36 | AG43 |
| 1 | Cellular Processes | Cell growth and death | 3936.127918 | 10845.68819 | 15671.10683 | 15357.91253 |
| 2 |  | Cell motility | 8114.739487 | 1895.362954 | 4467.630403 | 3801.07543 |
| 3 |  | Cellular community - eukaryotes | 60.667902 | 3507.702056 | 6479.089131 | 6654.409353 |
| 4 |  | Cellular community - prokaryotes | 23629.86499 | 742.416166 | 4185.636436 | 3284.888369 |
| 5 |  | Aging | 2644.925565 | 3509.27012 | 5041.188435 | 5037.163079 |
| 6 |  | Development and regeneration | 270.854436 | 1667.434966 | 3655.944142 | 4039.421672 |
| 7 | Environmental Information Processing | Environmental adaptation | 1760.393502 | 57733.99238 | 15961.78661 | 12289.61356 |
| 8 |  | Signal transduction | 26481.51804 | 23074.8865 | 31365.75489 | 31273.44476 |
| 9 |  | Signaling molecules and interaction | 67.320969 | 11.304246 | 156.397721 | 169.911963 |
| 10 |  | Folding, sorting and degradation | 11243.23978 | 14626.41723 | 16486.14597 | 15220.98191 |
| 11 |  | Membrane transport | 38292.70927 | 661.805194 | 5691.94095 | 3892.075533 |
| 12 | Genetic Information Processing | Replication and repair | 11253.05596 | 4950.475232 | 7111.582715 | 6746.900884 |
| 13 |  | Transcription | 1119.849072 | 9850.128817 | 7840.545787 | 6878.828053 |
| 14 |  | Translation | 20335.71722 | 20812.4234 | 22373.54953 | 19474.70728 |
| 15 | Human Diseases | Cancer: overview | 3317.436937 | 7370.438538 | 11580.84558 | 11601.24542 |
| 16 |  | Cancer: specific types | 1530.597185 | 3034.584519 | 4417.488783 | 4451.145182 |
| 17 |  | Infectious disease: bacterial | 5992.049075 | 9848.377436 | 12984.00081 | 12752.20936 |
| 18 |  | Infectious disease: parasitic | 284.613741 | 1508.068064 | 3198.073853 | 3373.465243 |
| 19 |  | Infectious disease: viral | 377.413137 | 16529.79787 | 19290.07439 | 18430.26829 |
| 20 |  | Neurodegenerative disease | 2798.53709 | 63951.29196 | 22648.55479 | 18824.22314 |
| 21 |  | Immune disease | 380.436413 | 2548.486472 | 1409.43626 | 1133.141227 |
| 22 |  | Cardiovascular disease | 3978.999915 | 57835.44873 | 15794.24693 | 12048.78555 |
| 23 |  | Endocrine and metabolic disease | 1202.787343 | 25239.10197 | 9401.829328 | 8461.297849 |
| 24 |  | Drug resistance: antimicrobial | 8528.808504 | 97.934941 | 1081.039025 | 748.693393 |
| 25 |  | Drug resistance: antineoplastic | 2380.908598 | 2750.258949 | 4701.731332 | 4834.779321 |
| 26 |  | Substance dependence | 105.787933 | 2357.201918 | 3786.856115 | 3899.29101 |
| 27 | Metabolism | Amino acid metabolism | 34317.51872 | 10320.72319 | 14889.90576 | 13061.68999 |
| 28 |  | Metabolism of cofactors and vitamins | 30624.8417 | 7810.165378 | 10407.5599 | 8401.904203 |
| 29 |  | Metabolism of other amino acids | 11811.22879 | 3772.827278 | 6021.891888 | 5347.228613 |
| 30 |  | Metabolism of terpenoids and polyketides | 5713.217432 | 1817.985671 | 2610.256592 | 2421.633774 |
| 31 |  | Lipid metabolism | 14218.55957 | 7670.173463 | 11970.23531 | 11602.371 |
| 32 |  | Glycan biosynthesis and metabolism | 11396.11576 | 4399.958326 | 6603.786326 | 5828.201536 |
| 33 |  | Energy metabolism | 26021.27978 | 60570.14771 | 17796.16278 | 12787.6932 |
| 34 |  | Carbohydrate metabolism | 46416.44206 | 12110.88478 | 20955.58547 | 18744.10222 |
| 35 |  | Nucleotide metabolism | 16980.67204 | 5157.149877 | 6025.312609 | 4900.75892 |
| 36 |  | Transport and catabolism | 1458.189202 | 15857.60055 | 22087.59243 | 21957.97633 |
| 37 |  | Xenobiotics biodegradation and metabolism | 10393.53321 | 2737.196973 | 3618.538958 | 3068.820382 |
| 38 |  | Biosynthesis of other secondary metabolites | 8275.725541 | 1581.647512 | 2954.11285 | 2133.446702 |
| 39 | Organismal Systems | Endocrine system | 2940.649604 | 6191.142168 | 10925.80463 | 11152.5603 |
| 40 |  | Immune system | 1182.609132 | 7417.208121 | 9850.512424 | 9470.508311 |
| 41 |  | Nervous system | 831.13662 | 32936.40938 | 12335.29809 | 10994.48019 |
| 42 |  | Sensory system | 19.953478 | 871.290509 | 2027.855007 | 2161.515849 |
| 43 |  | Excretory system | 171.268061 | 2647.514943 | 3329.159602 | 3314.916172 |
| 44 |  | Circulatory system | 258.010526 | 21554.18371 | 6763.676489 | 6166.958204 |
| 45 |  | Digestive system | 1037.360386 | 2223.542358 | 3651.401372 | 3769.967005 |
